# Gender differences in the association between pre-adolescent smoking initiation and emotional or behavioural problems

**DOI:** 10.1186/1471-2458-10-615

**Published:** 2010-10-18

**Authors:** Gea de Meer, Matty R Crone, Sijmen A Reijneveld

**Affiliations:** 1 Municipal Health Service Fryslân, Leeuwarden, the Netherlands; 2 Department of Health Sciences, University Medical Center Groningen (UMCG), University of Groningen, Groningen, the Netherlands; 3 TNO (Netherlands Organisation of Applied Scientific Research), Quality of Life, Leiden, the Netherlands

## Abstract

**Background:**

Emotional and behavioural problems are a risk factor for the initiation of smoking. In this study, we aimed to assess this relationship beyond clinical cut-off values of problem behaviour.

**Methods:**

Cross-sectional national survey among 9-13 year old children with data on smoking and Childhood Behaviour Checklist (CBCL) (N = 960). Relationships between smoking and tertiles of CBCL-scores were assessed.

**Results:**

Smoking was reported by 5.9% of the children (7.1% boys and 5.0% girls, P > 0.100). Relationships between smoking and problem behaviour were present in girls, but ot in boys. Among girls, smoking was associated with attention problems, thought problems, and delinquent behaviour. For attention problems and delinquent behaviour the associations were limited to the CBCL-scores in the uppermost 16% which agrees with the subclinical cut-off value.

**Conclusion:**

Pre-adolescent girls more frequently smoke if having attention problems, delinquent behaviour, or thought problems.

## Background

Smoking-related diseases are the most common cause of death in Western societies and affluent countries [1]. Almost all adult smokers started smoking during adolescence [2]. Starting smoking at young age has been shown a risk factor for daily smoking and dependence [3].

There is a great amount of evidence for the co-occurrence of smoking initiation and emotional or behavioural problems [4-9]. Therefore, smoking prevention might benefit from targeting children with problem behaviour. So far, however, it is unclear if smoking prevention would benefit most from improvement of psychosocial health of all children, or from a targeted approach on children with more severe behavioural problems. Previous studies mostly relied on a dichotomous definition of problem behaviour like a clinical diagnosis [4-7], or the clinical or subclinical score on the Childhood Behaviour Checklist (CBCL), as reflected by respectively the uppermost 10% and 15% scores in the population [8,9]. This, however, does not give information on CBCL-scores below the subclinical cut-off value.

In this community-based study, we studied the relationship between smoking initiation and problem behaviour beyond subclinical cut-off values among pre-adolescent elementary school children. In addition, we searched for gender differences in the relationship between behaviour problems and the uptake smoking. Previous studies have shown that girls more frequently take up smoking in early adolescence [10,11]. Gender differences in the association with problem behaviour may play a role, as suggested by Rohde et al [12]. Therefore, we studied relationships between pre-adolescent problem behaviour and the uptake of smoking for boys and girls separately.

## Methods

### Study protocol

The study was embedded in Preventive Child Healthcare (PCH) that provides health monitoring for all 0-19 year-old children according to Dutch public health legislation. Parents and their children are invited for regular examination that includes monitoring of development and growth, and screening activities (for example congenital disease, vision impairment, hearing loss). Participation rate to PCH routine examinations exceeds 90% without differences by gender, parental education, family income, and ethnic background [13].

Postal questionnaires were sent to a national sample of parent-child couples together with the invitation for a routine health examination by PCH. Parents completed questions on background characteristics, the child's physical health, and behaviour problems. The child questionnaire comprised data on smoking, bullying, and friends. Parents and children completed the questionnaires at home and delivered them in a sealed envelope at the PCH clinic when coming for the child's preventive health examination. The study protocol was approved by the local Medical Ethical Committee including verbal informed consent by parents to the child health nurse that took in the sealed questionnaires.

### Study population

The study population was obtained by means of a two-step procedure. First, a random sample of child-healthcare services was drawn, after stratification by region, urbanization ethnicity. Subsequently, for each PCH clinic a random sample of 100 children, grade 5-8 of primary school (age 7-13). After correction for stratified sampling, the samples were representative for the entire Dutch population in terms of income, type of family and parental educational level.

Out of the 1706 invited children 1350 (79%) parents gave informed consent for participation in the study. Most important reason for non-participation was a lack of interest in the study (37%). Children with missing data on sex (N = 46) or age (N = 4) were excluded. Since smoking did not occur among children under 9 years of age, we restricted the population to children aged 9 and older (N = 1230).

### Background characteristics

Parents completed questionnaire data on the child's and family's background characteristics including sex, family composition, ethnic origin, parental education, and family income.

### Smoking

Children were asked if they ever had smoked cigarettes, categorized as 'never', 'once or twice', 'regular', or 'daily'. For the current analysis we dichotomized the answers in 'never' and 'ever', since only 4 children reported 'regular' or 'daily' smoking.

### Child problem behaviour

Parents completed the 120 problem items of the validated Dutch version of the Child Behaviour CheckList (CBCL) [14,15]. For the current analysis, the CBCL smoking item was deleted. Raw CBCL scores were computed for eight syndrome scales, i.e. attention problems, aggressive behaviour, anxious/depressed, delinquent behaviour, social problems, somatic complaints, thought problems, and being withdrawn. Broadband groups of syndrome scales comprised internalizing (anxious/depressed, somatic complaints, withdrawn) and externalizing problems (aggressive or delinquent behaviour). All CBCL-scores were categorized in tertiles of the gender-specific distribution indicating three levels of problem behaviour.

### Statistical analysis

Statistical analyses were performed using the SPSS 12.0 statistical software package. Relationships between smoking initiation and tertiles of CBCL-scores were tested using Chi-square tests. Crude and multiple logistic regression analyses were performed. To explore differences by gender we performed stratified logistic regression analyses for sex.

## Results

Of the 1230 children aged 9-13 years, the CBCL was completed for 1118 children and additional data on smoking were available for 960 children (78.0%). Ever smoking was reported by 57 (5.9%) children. Children that reported ever smoking were older (P = 0.022) and less frequently had a highly-educated mother (P = 0.026) compared to never smokers. There was no difference between ever and never smokers for sex, ethnic background, family composition, family size, and father's education (table 1).

**Table 1:** Socio-demographic characteristics of smoking and non-smoking children

	All (N = 960)	Ever smoking		
			
		No (N = 903)	Yes (N = 57)	P-value
Gender, N = 960:				
Boy	468 (49%)	435 (48%)	33 (58%)	
Girl	492 (51%)	468 (52%)	24 (42%)	0.173
Age, N = 960:				
9-10 years	341 (36%)	329 (36%)	12 (21%)	
11-13 years	619 (64%)	574 (64%)	45 (79%)	0.022
Ethnic origin, N = 960				
Dutch	700 (73%)	653 (72%)	47 (82%)	
Non-Dutch	260 (27%)	250 (28%)	10 (18%)	0.123
Family composition, N = 946				
2 parents	849 (90%)	801 (90%)	48 (87%)	
1 parent	97 (10%)	90 (10%)	7 (13%)	0.494
Siblings, N = 960				
0 or 1	554 (58%)	518 (57%)	36 (63%)	
2 or more	406 (42%)	385 (43%)	21 (37%)	0.411
Mother's education, N = 931				
University or high vocational	198 (21%)	182 (21%)	16 (29%)	
Intermediate vocational	272 (29%)	251 (29%)	21 (37%)	
Low vocational or high school	461 (50%)	442 (50%)	19 (34%)	0.026
Father's education, N = 891				
University or high vocational	254 (26%)	239 (28%)	15 (29%)	
Intermediate vocational	242 (27%)	222 (27%)	20 (28%)	
Low vocational or high school	395 (44%)	378 (45%)	17 (33%)	0.289

Table 2 shows the distribution of smokers by tertiles of CBCL score. Among girls, a positive trend was observed for a report of smoking and a CBCL score for total problems (P = 0.062), delinquent behaviour (P = 0.050), attention problems (P = 0.010), and thought problems (P = 0.039). Except for thought problems, the higher prevalence rates only occurred for the uppermost tertiles of CBCL-scores. For boys we did not observe any relationships of smoking with CBCL-scores.

**Table 2:** Number of ever smokers by tertiles of the distribution for CBCL

	Tertile of CBCL score						
	
		Low	Intermediate		High		
				
	N	Ever smokers	N	Ever smokers	N	Ever smokers	p-value trend
	
Girls							
Total problems	170	6 (4%)	159	5 (3%)	163	13 (8%)	0.062
Externalizing problems	146	6 (4%)	190	7 (4%)	156	11 (7%)	0.211
Internalizing problems	194	8 (4%)	154	10 (6%)	144	6 (4%)	0.911
Aggressive behaviour	168	8 (5%)	179	6 (3%)	145	10 (7%)	0.412
Delinquent behaviour	245	8 (3%)	106	5 (5%)	141	11 (8%)	0.050
Anxious/depressed	133	4 (3%)	211	11 (5%)	148	9 (6%)	0.237
Somatic complaints	150	7 (5%)	178	7 (4%)	164	10 (6%)	0.544
Withdrawn	205	8 (4%)	120	6 (5%)	167	10 (6%)	0.352
Attention problems	189	4 (2%)	186	10 (5%)	117	10 (8%)	0.010
Social problems	162	10 (6%)	128	5 (4%)	125	7 (6%)	0.783
Thought problems	208	4 (2%)	126	10 (8%)	158	10 (6%)	0.039
**Boys**							
Total problems	163	9 (6%)	156	15 (10%)	149	9 (6%)	0.827
Externalizing problems	158	13 (8%)	167	11 (7%)	143	9 (6%)	0.508
Internalizing problems	155	11 (7%)	164	11 (7%)	149	11 (7%)	0.925
Aggressive behaviour	137	10 (7%)	182	13 (7%)	149	10 (7%)	0.845
Delinquent behaviour	158	9 (6%)	198	15 (8%)	112	9 (8%)	0.437
Anxious/depressed	129	10 (8%)	177	14 (8%)	162	9 (6%)	0.445
Somatic complaints	164	9 (6%)	185	13 (7%)	119	11 (9%)	0.227
Withdrawn	171	12 (7%)	182	13 (7%)	115	8 (7%)	0.990
Attention problems	141	10 (7%)	170	9 (5%)	157	14 (9%)	0.516
Social problems	150	11 (7%)	177	10 (6%)	141	12 (8%)	0.708
Thought problems	189	11 (6%)	113	11 (10%)	166	11 (7%)	0.737

Logistic regression analyses adjusted for age, sex and mother's education yielded statistically significant odds ratios for thought problems and delinquent behaviour (table 3). Gender stratified logistic regression were performed for associations with P < 0.100 in bivariate (table 2) or multivariate analyses (table 3), i.e. total problems, delinquent behaviour, attention problems and thought problems. In boys, none of the CBCL problem scores was associated with smoking initiation (data not shown).

**Table 3:** Odds ratios and 95% confidence intervals for tertiles of CBCL-scores; adjusted for age, mother's education, and sex.

	Tertile of CBCL-score		
	
	Low (reference)	Intermediate	High
Total problems	1.0	1.4 (0.7;2.9)	1.9 (09;3.7)
Externalising problems	1.0	0.8 (0.4;1.7)	1.3 (0.7;2.5)
Internalising problems	1.0	1.2 (0.6;2.4)	1.2 (0.6;2.4)
Aggressive behaviour	1.0	0.9 (0.5;1.9)	1.3 (0.7;2.5)
Delinquent behaviour	1.0	1.7 (0.8;3.3)	2.2 (1.1;4.4)
Anxious/depressed	1.0	1.1 (0.6;2.1)	1.2 (0.6;2.5)
Somatic complaints	1.0	1.2 (0.6;2.2)	1.4 (0.7;2.8)
Withdrawn	1.0	1.1 (0.6;2.1)	1.7 (0.8;3.3)
Attention problems	1.0	1.6 (0.8;3.1)	1.8 (0.9;3.5)
Social problems	1.0	1.0 (0.4;2.3)	1.4 (0.8;2.6)
Thought problems	1.0	2.8 (1.4;5.5)	1.7 (0.8;3.3)

In girls, taking up smoking occurred more frequently in girls with a higher CBCL problem score for thought problems, attention problems, or delinquent behaviour (table 4).

**Table 4:** Odds ratios and 95% confidence intervals for tertiles of CBCL-scores in girls; adjusted for age and mother's education.

	Tertile of CBCL-score		
	
	Low (reference)	Intermediate	High
Total problems	1.0	0.9 (0.3;3.1)	2.7 (1.0;7.5)
Delinquent behaviour	1.0	1.6 (0.5;4.9)	2.8 (1.1;7.3)
Attention problems	1.0	3.1 (0.9;10.3)	3.6 (1.1;11.8)
Thought problems	1.0	4.5 (1.4;14.9)	3.7 (1.1;12.1)

**Figure 1:**
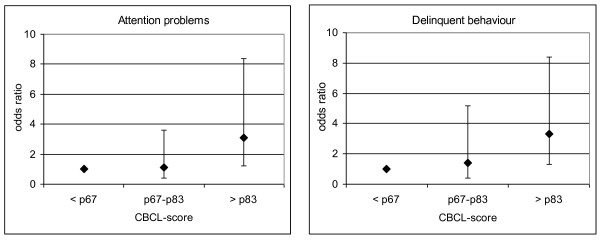
**Odds ratios and 95% confidence intervals for the relationship between smoking and CBCL-scores in girls; adjusted for age and mother's education**.

Aiming at a more precise assessment of the relationships within the uppermost tertile of CBCL scores for attention problems and delinquent behaviour, logistic regression was repeated for split groups of the uppermost tertile (67^th ^- 83^rd ^and >83^rd ^percentiles). To increase statistical power, the lowest and intermediate tertiles were combined as the reference group. As shown in Figure 1, for attention problems and delinquent behaviour only the uppermost 16.7% values of CBCL-scores were associated with ever smoking (P < 0.050).

## Discussion

In this study, we found that among pre-adolescent girls smoking initiation was associated with delinquency, attention problems, and thought problems. For delinquency and attention problems, this was limited to CBCL-scores in the subclinical and clinical range. In contrast, the association of smoking initiation with thought problems applied to mild problems as well. In boys smoking initiation was not associated with problem behaviour.

Our results confirms a relationship of smoking with attention deficit [16,17], conduct problems [4,7,18,19], delinquency and thought problems [8]. To our knowledge this is the first study that took into account the distribution of the data on child behaviour by analyzing in tertiles. Theoretically, this provides information on the role of problem behaviour beyond clinical cut-off values. Interestingly, our final results show that an association with smoking conferred the uppermost 16.7% of CBCL scores, i.e. close to the subclinical cut-off value of the uppermost 15% CBCL scores [14,15].

Our results thus suggest that smoking prevention will benefit most from targeting a 'high-risk' group of children with behavioural problems. The association of smoking initiation with specific domains of problem behaviour may be taken into consideration as an additional reason for a 'high-risk' group approach. This may be added to general population strategies for smoking prevention, thereby increasing its effect in the subgroup of children with behavioural problems. To our opinion, this is a relevant group for smoking prevention since early starters have been shown at high risk of regular use, dependence and abuse later in life [6,20].

In this study, we did not collect data on possible explanatory factors for the relationship between behavioural problems and cigarette smoking. Peer group pressure may play a role in children with delinquent behaviour [21]. In children with attention problems, self-medication may contribute to the development of nicotine dependence since tobacco is known to ameliorate symptom severity, and enhance general attention [22-24]. Self-medication may also apply to thought problems as shown in schizophrenic patients [25,26]. We, however, emphasize that 'thought problems' in this study is not synonymous with psychiatric pathology. CBCL items for thought problems address problems with reality testing and obsessive/compulsive behaviour, though without a relationship with DSM-diagnoses. Since we did not collect data on any action to relieve symptoms, explanations with respect to self-medication for our results should be considered speculative. Nevertheless, our results are in agreement with previous studies that showed an earlier initiation of smoking in subjects with attention deficit or thought problems including schizophrenia [12,27,28]. It might be that children with symptoms of attention deficit or thought problems search wittingly or unwittingly for relieve of symptoms, for which smoking is rather easy available. Follow-up studies are needed to get insight in the mechanisms of smoking initiation and continuation in children with emotional or behavioural problems.

Interestingly, associations between smoking and psychosocial problems were observed in girls only, and not in boys. We do not have a clear explanation for this finding, though it confirms results of other studies that similarly found a stronger association between smoking and psychopathology in girls [4,12,17]. Selective refusal to report smoking is unlikely, neither among boys with problem behaviour nor among girls without problem behaviour. Similarly, it is unlikely that parents of smoking girls were more likely to identify problem behaviour by CBCL compared to parents of smoking boys. An explanation of our gender dependent findings may be a larger proportion of girls having started puberty at age 8-12 years compared to boys, with a concurrent rise in behavioural and emotional problems among girls.

The main limitation of our study is the small number of children that smoked. The potential for further interpretation is limited due to lack of statistical power and allow statistical analysis taking into account multiple confounders. In this study, we therefore limited multiple logistic regression analyses to age, gender, and variables for which frequencies differed statistically significant between smokers and non-smokers, i.e. mother's education. Due to the low number of regular smokers, we limited statistical analyses to starters. In this study, we relied on a self-report of smoking. One might wonder if this introduces information bias. Though, previous studies have shown good validity for self-report of smoking [29].

For the current analyses, data were available for 78.0% of the 79.0% parent-child couples that agreed to participate in the study (i.e. 62% of the invited population). We do not think this has importantly influenced our results, since participants and non-participants did not differ in background characteristics.

Causal inference from our study results is limited due to the cross-sectional design which does not allow inference on the order of smoking and behavioural problems. From previous follow-up studies we assume that psychopathology precedes smoking in at least a substantial proportion of adolescents [4,5,9,12]. Large-scale, long-term follow-up studies from early childhood to adulthood will unravel etiological relationships between behavioural problems in smoking trajectories of experimentation, continuation, and regular use.

In summary, in this study we found that the uptake of smoking in pre-adolescent and early adolescent girls occurred more frequently in those with a higher level of psychosocial problems as reported by their parents. Since the relationship was present only for the uppermost 16.7% of CBCL scores, which agrees with subclinical and clinical scores. Our results suggest that girls in their early teens with problem behaviour may benefit from targeted smoking prevention.

## Conclusion

Among pre-adolescent girls, aged 8-13 years, problem behaviour is associated with the uptake of smoking. Among boys of this age, smoking was not associated with problem behaviour.

## Competing interests

The authors declare that they have no competing interests.

## Authors' contributions

GM conceived the research question for this paper, performed statistical analyses, and wrote the paper. MR participated in the study design and protocol, supervised data collection and data management. SAR was the principle investigator of this study and conceived the study and financial support. The final manuscript was discussed with all authors, and edited as recommended by co-authors. All authors read and approved the final manuscript.

## Pre-publication history

The pre-publication history for this paper can be accessed here:

http://www.biomedcentral.com/1471-2458/10/615/prepub
